# Geckos differentiate self from other using both skin and faecal chemicals: evidence towards self-recognition?

**DOI:** 10.1007/s10071-023-01751-8

**Published:** 2023-02-08

**Authors:** Birgit Szabo, Eva Ringler

**Affiliations:** Division of Behavioural Ecology, Institute of Ecology and Evolution, University of Bern, 3032 Bern, Switzerland

**Keywords:** Chemical discrimination, Communication, Reptile, Social cognition, Squamate

## Abstract

Self-recognition is the ability to recognise stimuli originating from oneself. Humans and some non-human animals show evidence of true visual self-recognition in the mirror test. They use their reflection to inspect themselves and to remove a mark that is only visible in the mirror. Not all animals, however, rely primarily on vision. In lizards, chemical cues are crucial in social interactions, and therefore, lizards would benefit from a chemical self-other distinction. Here, we test the tokay gecko (*Gekko gecko*), a social species, on their ability to discriminate their own skin and faecal chemicals from those of same-sex, unfamiliar conspecifics. We predicted that individuals would show more self-directed behaviour when confronted with the chemicals from unfamiliar individuals within their home enclosure as a sign of the need for increased comparison. Geckos showed higher self-directed responses towards chemicals from unfamiliar individuals compared to self-produced chemicals and a water control. Furthermore, scat and skin chemicals (regardless of origin) elicited similar but stronger responses than peppermint oil pointing towards a possible social function of scat piles. Although further tests and controls are needed, our study provides evidence towards chemical self-recognition and for a social function of scat piling in tokay geckos.

**Supplementary Information:**

The online version contains supplementary material available at 10.1007/s10071-023-01751-8.

## Introduction

Self-recognition is the ability to recognise cues that represent/originate from oneself (visual images, olfactory cues, acoustic stimuli) (Gallup 1970; Gallup et al. 2011; Platek et al. 2004). Research into self-recognition aims to uncover self-awareness (the ability to become the object of one’s own attention) and its emergence across humans and non-human animals (Gallup 1970; Gallup et al. 2011). The method of choice is the mirror self-recognition (MSR) test. In this test, a subject is confronted with a mirror and provided with a mark that can only be seen using the reflection in the mirror. Confirmation of MSR occurs when the subject spontaneously inspects the mark and attempts to remove it using their reflection (Gallup 1970). A number of important control conditions need to be implemented. First, individuals need to show spontaneous self-directed behaviours such as inspecting themselves in front of the mirror (Gallup 1970; Gallup and Anderson 2020). Second, an invisible mark has to be used to exclude that physical irritation caused by the mark itself or the process of marking is triggering the behaviour (Gallup 1970). Third, a mark has to be applied in a spot that can be seen without the use of the mirror to confirm the subjects’ motivation to remove marks in general (Gallup and Anderson 2018). Humans, most great apes, elephants, dolphins and cleaner fish show MSR (Gallup 1970; Gallup et al. 2011; Kohda et al. 2019; 2022; Loth et al. 2022; Plotnik et al. 2006; Reiss and Marino 2001). The evidence from species other than humans and great apes has, however, led to controversial discussion (Gallup and Anderson 2018; 2020).

Not all species primarily depend on their visual sense. This has led to the development of the sniff-test for self-recognition used in dogs whose primary sense is smell (Cazzolla Gatti 2016). These studies have demonstrated that dogs discriminate between their own odour and that of conspecifics. They sniff the urine of unfamiliar dogs for longer than their own urine (Cazzolla Gatti 2016; Horowitz 2017). Furthermore, they sniff their own odour longer when it is modified than the chemical used for modification by itself (Horowitz 2017). Nonetheless, some researchers have criticised these studies as not being a true equivalent to the MSR test because dogs do not show self-directed behaviour in the sniff-test which is an important control in the MSR test even without a mark present (Gallup and Anderson 2018; 2020).

Reptiles also rely strongly on chemicals (i.e. pheromones) when it comes to individual recognition, territoriality, social interactions and mate choice (Norris and Lopez 2001). Consequently, chemical self-recognition tests are fairly common in squamates which include lizards (e.g. Aguilar et al. 2009; Alberts 1992; Aragón et al. 2001; Bull et al. 2000; Cooper 1999; Graves and Halpern 1991; López et al. 1997; Vicente and Halloy 2018), snakes (e.g. Burghardt et al. 2021; Chiszar and Smith 1991; Halpin 1990) and amphisbaenids (e.g. López et al. 1997). In lizards, pheromones might originate from the skin or specialised glands such as femoral glands which are most pronounced in males (Norris and Lopez 2001). Many species also possess cloacal glands that deposit pheromones onto the faeces (Norris and Lopez 2001). This is especially important in scat piling lizards which defecate repeatedly in the same location (Bull et al. 1999a). Similar to latrines in mammals (e.g. Green et al. 2015; King et al. 2017), these scat piles can have a social function by communicating, for example, territory ownership (Bull et al. 1999a; 1999b) and group identity (Bull et al. 2000; but see Shah et al. 2006). Lizards detect pheromones using tongue-flicks (TF), protrusions of the tongue forward towards a stimulus (e.g. on the ground or on a swab) to collect chemicals (Cooper 1994), and generally, show increased TF rates towards stimuli from unfamiliar conspecifics (e.g. Alberts 1992; Aragón et al. 2001; Cooper et al. 1999; Graves and Halpern 1991). Although rarely considered in lizards, self-directed TF were shown by male desert iguanas (*Dipsosaurus dorsalis*) after detection of their own femoral gland secretions but not in response to the secretions of unfamiliar males (Alberts 1992). Compared to what was found in dogs (Cazzolla Gatti 2016; Horowitz 2017), these results demonstrate more conclusive evidence for self-recognition using chemicals although further test are needed.

Among lizards, some gecko species demonstrated the ability to discriminate between self-produced chemicals and chemicals from unfamiliar, same-sex conspecifics (Carpenter and Duvall 1995). Some gecko species scat pile which suggests either a communicative function aimed at conspecifics, an anti-predatory function to avoid detection of refuges or both (Bull et al. 1999a; Carpenter and Duvall 1995; Shah et al. 2006). Geckos are, therefore, an excellent model to investigate chemical self-recognition and gain insights into the social function of different pheromones (originating from the skin and faeces). Here, we test the tokay gecko (*Gekko gecko*), a large (up to 185 mm Snout Vent Length), nocturnal, insectivorous, social (territorial and family living) and scat piling gecko species from tropical South-East Asia (Grossmann 2006). The aims of this study were to

1. Investigate if tokay geckos discriminate between self-produced chemicals and chemicals produced by unfamiliar, same-sex conspecifics on cotton swabs (Cooper 1998). We predicted that geckos would show increased responses towards the odour originating from unfamiliar individuals (Alberts 1992; Cooper et al. 1999; Graves and Halpern 1991).
2. Investigate if geckos show behaviour indicative of comparison between their own chemicals and those originating from unfamiliar, same-sex conspecifics. Since tokay geckos are territorial, show site fidelity and scat pile (Grossmann 2006), it is likely that they deposit chemicals to mark their territory/ home range. Consequently, their familiar surroundings are saturated with their own odour and by testing them within their home enclosure it is possible to detect “self-directed” behaviour through ground-directed TF. We expected to find both ground- and swab-directed TF (tongue-flicks) as a sign of comparison between self and other. We predicted, however, less ground-directed responses (sampling their own odour; i.e. self-directed behaviour) when confronted with their own odour as it is familiar.
3. Investigate if faecal chemicals have a similar function to skin chemicals and predicted that scat chemicals were as effective as skin chemicals in eliciting a response showing that scats have a communicative function.

## Methods

### Study animals, housing and husbandry

We tested 22 captive bred, adult tokay geckos, 10 males and 12 females (male snout vent length (SVL): range = 11.35–15.02 cm, female SVL: range = 11.29–13.72 cm). Sex was determined by the presence (male) and absence (female) of femoral glands (Grossmann 2006). Animals were acquired from different breeders across Europe and approximately 2–6 years old at the time of the study.

Geckos are kept singly in terraria (females–45 L × 45 B × 70 H cm; males–90 L × 45 B × 100 H cm) in a bioactive setup. Gecko enclosures (made of rigid foam slabs with a glass front) are equipped with a drainage layer of clay pebbles and a layer of organic rainforest soil (Dragon BIO-Ground) on top separated by a mosquito mesh to prevent mixing of the layers. On the soil surface, we spread autoclaved red oak leaves. Collembola, isopods and earthworms in the soil break down the faecal matter produced by the geckos. Each enclosure also includes a compressed cork back wall, cork branches, refuges made out of cork branches cut in half (hung on the back wall with hooks) as well as plants. Enclosures are located in a fully climate controlled environment under a reversed photo-period. Environmental temperature ranged from approximately 25 °C at night to 30 °C during the day. In addition to the room light, each enclosure is equipped with an UVB light (Exo Terra Reptile UVB 100, 25 W). We also provide a heat mat for thermoregulation (TropicShop; increase of ~ 5 °C). Humidity was set to 50% which is briefly increased to 100% by daily rainfall (osmotic water) twice for 30 s every 12 h at 5 pm and 4 am. Enclosure temperature is recoded automatically to an accuracy of 0.1 °C by the system responsible for regulating the environment within rooms. All enclosures are set up on shelves with small enclosures on the top and large enclosures on the bottom and animals are spread evenly across two rooms separated by a small hallway.

Lizards are fed three times per week on Monday, Wednesday and Friday with 3–5 adult, gut loaded (reptile planet LDT cricket mix, Purina Beyond Nature’s Protein^™^ Adult dry cat food and fresh carrots) crickets (*Acheta domesticus*) to provide optimal nutrition (Vitamin D and calcium). We feed geckos using 25 cm long metal forceps to monitor their food intake closely. Lizards have access to water ad libitum. All individuals that were used in this study were naïve to the experimental procedure.

### Experimental setup and stimuli

Lizards were tested in their home enclosures (between 10th of August and 11th November 2021) to reduce stress of handling (Langkilde and Shine 2006) and enable us to measure self-directed behaviour. Testing was conducted under red light (PHILIPS TL-D 36W/15 RED). The light we use has a red component at 718 nm which is not detectable by the tokay geckos’ photoreceptors (Loew 1994). Furthermore, a blue UV-C component at 282 nm is also produced which is visible to the geckos (Loew 1994) and promotes gecko activity (personal observation).

Since animals were spread across two rooms, each room was tested on a different, non-feeding day (either Tuesday or Thursday) once a week for a total of six repetitions (three for skin and three for scat chemicals). The order in which individuals were tested, stimuli (controls, own, same-sex unfamiliar) and treatment (skin, faeces—i.e. scat) were randomised across days. Each animal was tested with 4 stimuli: (1) the odour of a moist (tap water) paper towel (within treatment control), (2) peppermint essential oil (farfalla AromaCare) on a moist paper towel (between treatment control), (3) their own odour either from skin or scat (own) and (4) the odour from skin or scat of a same-sex individual from the other room (unfamiliar). The water control was used to ensure that responses were consistent across treatments and time. The peppermint control was used to exclude novelty as a cause for increased responses.

To create the control stimuli (water and peppermint oil), one side of a cotton swab was taped 10 times on a moistened paper towel (with or without peppermint oil). As the familiar odour, we used the individuals own odour either from their skin collected by gently rubbing one side of a cotton swab over its back and/or sides 10 times or from a fresh (no older than 2 days) scat. Lizard skin and scat chemicals were collected using dry swabs to ensure that lipids were collected (Bull et al 1999b). The cotton swab was rubbed on the scat until a stain was visible. To create the same-sex unfamiliar stimulus, we took chemicals from the skin or scats of a same-sex individual from the second room. Although animals never had direct contact with each other within a room, we were unsure if the smell of individuals could spread within a room. To ensure a high degree of unfamiliarity, we used the individuals from the second room located across a small hallway. The same methods as for collecting individuals own odour was used. Each individual was tested on their reaction towards the odour of three different same-sex conspecifics (three trials). From each conspecific, both chemicals from skin and faeces were used to be able to compare the reaction across treatments while controlling for identity. All cotton swabs were marked at the back to indicate on which side the stimulus was applied. This was done so the experimenter could present each cotton swab with the stimulus facing downwards to exclude the use of visual information originating from faeces or UV-reflecting chemicals (Mason 1992).

### Experimental procedure

Control stimuli were set up first, then all swabs with lizards own odour, and lastly, all swabs with the unfamiliar odour. This ensured 20–30 min between stimulus collection and test of focal individuals. All swabs were placed in clay bowls in the order of presentation (electronic supplementary material Figure S1). We setup only half of the individuals at a time to prevent excessive degradation of chemical stimuli. After testing, the clay bowls were thoroughly cleaned with hot water and a sponge and dried upside down. The experimenter ensured that the inside of the bowls was never touched and that the cotton swabs within a bowl never came in contact. After setup, we first tested all individuals with the first cotton swab, then the second and finally with the third. This ensured 10–15 min between stimulus presentations and reduce carry-over effects.

At the start of a trial, we placed a dim white light (LED, SPYLUX^®^ LEDVANCE 3000 K, 0.3 W, 17 lm) necessary to record lizard behaviour on top of the enclosure. Next, we located an individual in its enclosure (gently removing the refuge if necessary). Then, a cotton swab was presented holding it in a pair of 25 cm long metal forceps to prevent the experimenters’ odour interfering. The experimenter was visible during trials. Trials were recorded on video (GoPro Hero 5 or Samsung S20). By the second week of testing, we detected a large decrease in bites likely caused by lizards learning that the cotton swab was not edible. We, therefore, decided to repeat the first trial at the end of the testing period to ensure that our measurements were not confounded by changes in behaviour.

### Data collection

Videos were analysed blind as to which stimulus was presented. We used VLC media player (Version 3.0.7.1, Vetinari, Intel 64 bit) to score behaviour shown during trials. We scored bites, TF, gular pumping (Norris and Lopez 2011), deep breaths, and turns (Table 1; electronic supplementary material video M1). TF were divided into swab (tongue tip pointing in the direction of the swab) and ground (head down, tongue tip pointing in the direction of the ground) directed TF to record the comparison between the presented stimuli (swab) and own odour (ground). We measured trial time (s) starting from the time the stimulus was presented within 1 cm of the lizards’ snout until either 120 s without a bite or TF elapsed, the lizard performed a turn or 60 s after the first bite or TF (Aragón et al. 2001; López et al. 1997; Martin et al. 2020). Furthermore, enclosure temperature was recoded automatically to an accuracy of 0.1 °C by the system responsible for regulating the environment within rooms. In addition, 35% of videos were scored by an independent observed who was unaware of the objectives of the study. We calculated inter-observer reliability using spearman rank correlation and found reliability was generally high (swab-directed TFs: *r*_s_ = 0.706, *p* value < 2.2 × 10^–16^, *S* = 162,090; ground-directed TFs: *r*_s_ = 0.871, *p* value < 2.2 × 10^–16^, *S* = 71,184; bites: *r*_s_ = 0.952, *p* value < 2.2 × 10^–16^, *S* = 27,310; gular pumping: *r*_s_ = 0.901, *p* value < 2.2 × 10^–16^, *S* = 2323.1; deep breaths: *r*_s_ = 0.759, *p* value < 2.2 × 10^–16^, *S* = 62,613).

**Table 1:** Ethogram of behaviours shown by tokay geckos in response to chemical stimuli

Name of behaviour	Description
Gular pumping	Regular breathing. One up and down movement of the lizards’ throat = 1 breath. Only visible from the ventral side. Recorded as counts
Deep breath	One extension and retraction of the flanks behind the front legs. Visible from the dorsal and ventral side. Recorded as counts
Tongue-flick directed at the swab	Quick protrusion of the tongue forward away from the mouth with the tip directed at the swab. NOT licking of the lips which is also a protrusion of the tongue but along the skin of the mouth. Recorded as counts
Tongue-flick directed at the ground	Quick protrusion of the tongue forward away from the mouth while the head is tilted downwards directing the tip of the tongue towards the ground. Not licking of the lips which is also a protrusion of the tongue but along the skin of the mouth. Recorded as counts
Bite	The swab is taken between the upper and lower jaw. May be accompanied by shaking of the head. Recorded as counts
Turn	The lizard moves away from the swab. The whole body moved either past the swab, backwards away or involved a turn away from the swab. Recoded as yes or no. A trial was terminated if this behaviour was shown

### Statistical analysis

#### Power analysis

Before data collection, we performed a power analysis using G*power (Faul et al. 2007; 2009). As our study was designed as a 2 × 2 × 3 factorial designed, we calculated power based on a within factor repeated measures ANOVA. The literature on chemical discrimination in other lizard and worm lizard species (Alberts 1992; Cooper et al. 1999; López et al. 1997) generally suggested large effect sizes. We were, however, unsure what effect size to expect from our geckos and therefore calculated the minimal effect size that could be reliably detected at a power of 0.8. We specified an alpha level of 0.05, a power of 0.8, six groups with three measurements, a correlation among repeated measures of 0.5 and a correction of 1. With these settings and a sample size of 24 individuals we are able to detect an effect size of 0.3 at an actual power of 0.99. The sample size used in our study was 22 individuals. We expected, however, only a slight reduction in the actual power to detect a small effect size.

#### Data analysis

We used generalised linear mixed zero-inflation negative binomial models (GLMM, package glmmTMB, Brooks et al. 2017) due to the large amount of zero TF in our data. First, we analysed if TF towards water (response variable) differed across treatments (peppermint oil, scat or skin); room (room 2 or 5), sex (male or female), animal test order and temperature were also included as fixed effects. Animal identity was included as the random effect. As we only found a strong effect of sex (electronic supplementary material Table S1), we analysed if the total TF produced were influenced by treatment, room, the order in which stimuli were presented or animals were tested, trial and temperature (fixed effects) in females only. Again, animal identity was included as the random effect. We were also interested if the difference in size between the test subject and the unfamiliar individual (delta SVL) from which the odour was taken affected TF. To this end, we looked at TF produced by females in the unfamiliar condition only as the response variable in another zero-inflation negative Binomial model. We included TF as the response variable, delta SVL as the fixed effect and animal identity as the random effect.

For the two measures of breathing (gular pumping and deep breaths), we first divided the number of breaths by the trial time to get a comparable measure (gular pumping and deep breaths per second). We used gular pumping (i.e. regular breaths) and deep breaths per second as the response variable in linear mixed effects models (LME, package lmerTest, Kuznetsova et al. 2017) with Gaussian family including treatment, stimulus, sex, stimulus order, trial, temperature and room as fixed effects. Both models conformed to the assumption of residual normality (visual inspection of qqplots). Both models included a random effect of animal identity and session to account for repeated measures. We did not analyse bites because they were shown too infrequent to be analysed.

To identify if females compared their own odour to that of an unfamiliar individual, we analysed swab- and ground-directed TF shown within the same trial across stimuli (skin and scat pooled). TF were used as the response variable and direction of the TF (ground or swab) in interaction with stimulus source (water control, own or unfamiliar odour), as well as temperature were included as the fixed effects and we included trial and animal identity as random effects. We compared TF produced in the different treatments as well as ground- and swab-directed TF across stimulus sources using least-square means (LSM, Lenth 2021).

Data analysis was done with R (Version 4.0.3; R Development Core Team 2020). All zero-inflation models included an offset of trial time to account for differences in trial length, a dispersion component of session and accounted for zero inflation based on treatment, sex and animal identity where appropriate. We report our results following Muff and colleagues (Muff et al. 2022): *p* > 0.1 no evidence, 0.1 < *p* < 0.05 weak evidence, 0.05 < *p* < 0.01 moderate evidence, 0.01 < *p* < 0.001 strong evidence, *p* < 0.001 very strong evidence.

## Results

One female (G015) could not be tested as she was too anxious and was only used as a stimulus donor. All other geckos habituated fast to being rubbed on their back with a swab and did not flee during stimulus collection by the second week of testing (the first week of testing was not used for analysis).

### General reaction towards the different stimuli

**Fig. 1:**
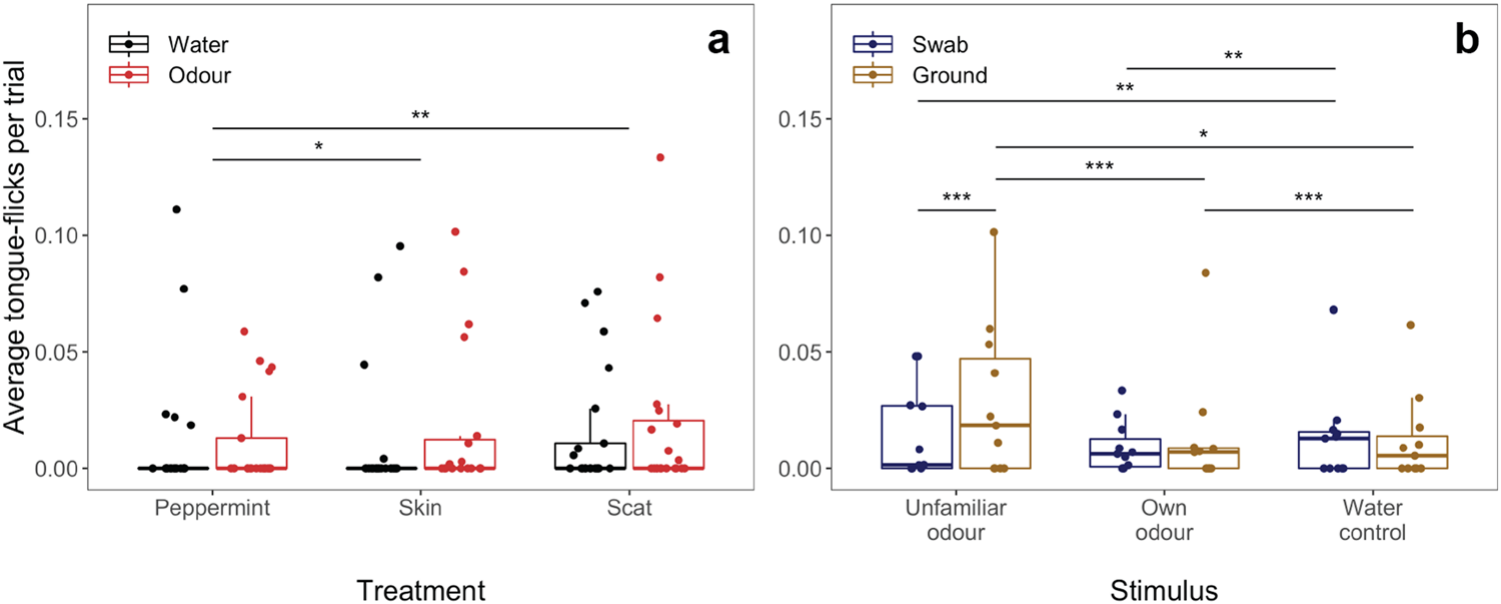
Box plots showing results of TF towards different stimuli in female geckos. **a** Average TF produced in the three treatments (peppermint control, skin and scat chemicals) split between responses towards tap water (black) and odour (red). **b** Average TF produced across the presented stimuli separated into responses towards the swab (blue) and the ground (brown). The bold line indicates the median, the upper edge of the box represents the upper quartile, the lower edge the lower quartile, the whisker the maximum and minimum, dots represent individual data. * *p* < 0.05, ** *p* < 0.01, *** *p* < 0.001 (color figure online)

**Fig. 2:**
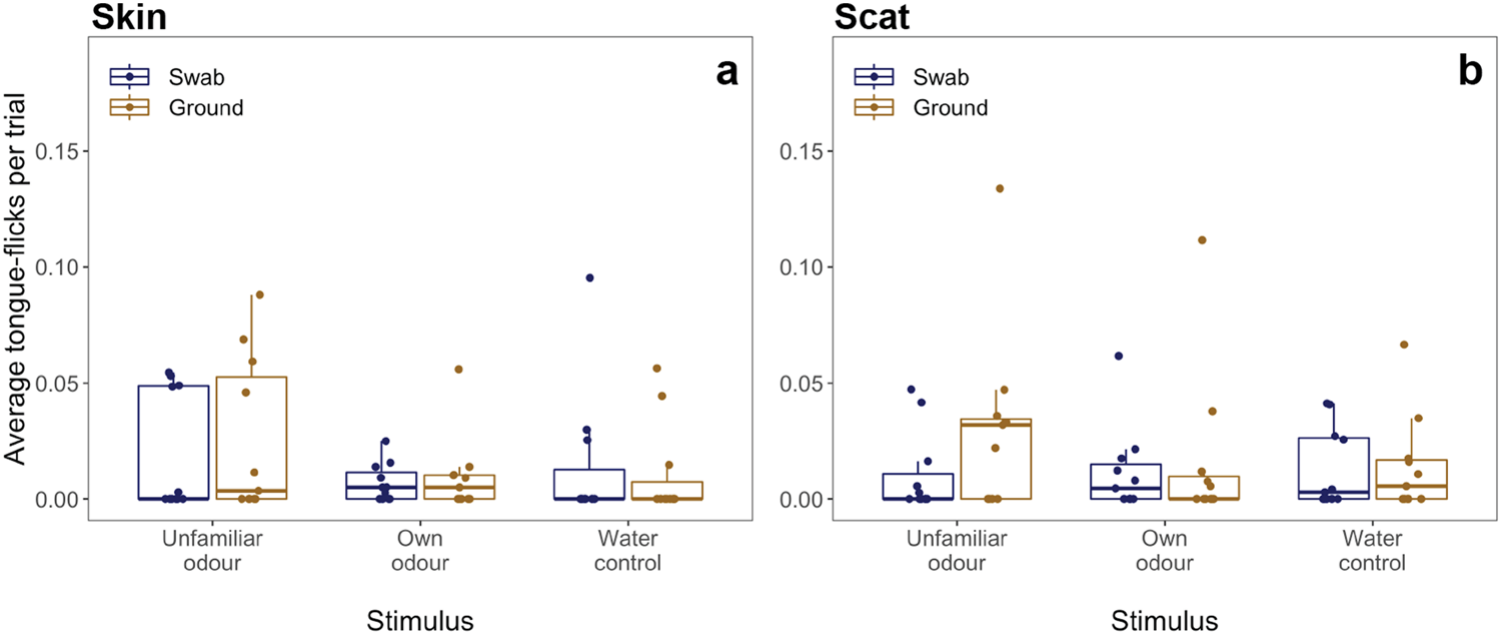
Box plots showing results of TF towards different stimuli separated in responses towards skin and scat chemicals in female geckos. **a** Average TF produced in response to skin sourced stimuli separated into responses towards the swab (blue) and the ground (brown). **b** Average TF produced in response to scat sourced stimuli separated into responses towards the swab (blue) and the ground (brown). The bold line indicates the median, the upper edge of the box represents the upper quartile, the lower edge the lower quartile, the whisker the maximum and minimum, dots represent individual data (color figure online)

We found no evidence that lizards differed in their reaction towards water across treatments (LSM, peppermint-skin = − 26.43, standard error = 27.79, *t* ratio = − 0.951, *p* value = 0.609; peppermint-scat = − 30.77, standard error = 23.92, *t* ratio = − 1.286, *p* value = 0.405; skin-scat = − 4.34, standard error = 8.18, *t* ratio = − 0.53, *p* value = 0.857; electronic supplementary material Table S1). We found strong/moderate evidence (respectively) that, compared to the peppermint oil, lizards tongue-flicked more in the scat (LSM, peppermint-scat = − 18.92, standard error = 5.98, *t* ratio = − 3.165, *p* value = 0.005; Fig. 1A) and skin treatment (LSM, peppermint-skin = − 17.03, standard error = 7.14, *t* ratio = − 2.385, *p* value = 0.047; Fig. 1A). We found no evidence for a difference in TF produced in the skin and scat treatment (LSM, skin-scat = − 1.88, standard error = 2.99, *t* ratio = − 0.629, *p* value = 0.804; Fig. 2). We found strong evidence that males tongue-flicked less than females (GLMM, estimate_male_ = − 47.915, CI_low_ = − 82.139, CI_up_ = − 13.961, *z* value = − 2.744, *p* value = 0.006) and that lizards tongue-flicked less at higher temperatures (electronic supplementary material Table S1, S2, S6). We found no evidence that the size difference to the stimulus individual affected TF (GLMM, estimate_deltaSVL_ = − 0.034, CI_low_ = − 2.184, CI_up_ = 2.117, *z* value = − 0.031, *p* value = 0.976, electronic supplementary material Table S3). We found no significant effects of any of the fixed effects on gular pumping or deep breaths per second (electronic supplementary material Table S4 and S5).

### Ground- versus swab-directed tongue-flicks

We found very strong evidence that females produced more ground- than swab-directed TF when unfamiliar odour was presented (LSM, swab-ground = − 33.970, standard error = 4.950, *t* ratio = − 6.860, *p* value = 0.0001; Fig. 1B). We found strong evidence that females directed more swab TF towards tap water than their own (LSM, water-own = 19.2, standard error = 5.76, *t* ratio = 3.339, *p* value = 0.003; Fig. 1B) and unfamiliar conspecific odour (LSM, water-unfamiliar = 20.7, standard error = 6.01, *t* ratio = 3.447, *p* value = 0.002; Fig. 1B; electronic supplementary material Table S6). Furthermore, we found very strong/moderate evidence (respectively) that more ground-directed TF were produced towards water than own odour (LSM, water-own = 17.2, standard error = 4.64, *t* ratio = 3.71. *p* value = 0.0007; Fig. 1B) and towards unfamiliar conspecific odour than water (LSM, water-unfamiliar = − 12.9, standard error = 4.73, *t* ratio = − 2.735, *p* value = 0.018; Fig. 1B) and own odour (LSM, own-unfamiliar = − 30.1, standard error = 5.28, *t* ratio = − 5.709, *p* value = 0.0001; Fig. 1B; electronic supplementary material Table S6). We did not detect any significant zero inflation or over-dispersion (electronic supplementary material Tables S1, S2, S3) except in the model comparing ground- and swab-directed TF where session was significant in the dispersion model (electronic supplementary material Table S6).

## Discussion

Across treatments, geckos responded similarly to water and responded stronger to social stimuli (skin and scat chemicals) than peppermint oil. Females showed both swab- and ground-directed TF. We found very strong evidence that females directed more TF to the ground than the swab when presented with chemicals from unfamiliar conspecifics. We found evidence that females produced more ground-directed TF in response to unfamiliar conspecific odour than their own or water and produced more ground TF in response to water than chemicals originating from themselves. Only females tongue-flicked reliably; males tongue-flicked a total of three times.

Based on previous studies in other lizards (e.g. Alberts 1992; Cooper et al. 1999; Graves and Halpern 1991), we predicted that tokay geckos would show more TF towards chemical stimuli originating from unfamiliar, same-sex conspecifics. Our results are in line with these studies but only in females. Males only tongue-flicked a total of three times during the course of the experiment. Either, males do not rely as strongly on skin and scat chemicals for individual recognition, they show a delayed response which we did not record using our methodology or the presence of the experimenter had a stronger effect on male behaviour than on the behaviour of females. We observed an increase in activity including TF in some males after trials had ended, although only by accident late into the experiment as the low light conditions (red light) prevented observations of behaviour after removal of the white light. Male tokay geckos are territorial (Grossmann 2006) and their behaviour might be interpreted as searching for the intruder. It is, however, necessary to run additional tests recording not just the immediate response of males within 2 min but record behaviour for a longer time such as 10–15 min after stimulus presentation. Furthermore, males might react stronger to femoral gland secretions similar to male amphisbaenians (*Blanus cinereus*; Cooper et al. 1994) which should be tested in the future. Finally, changing the testing procedure to remove the experimenters’ presence should also be considered in future tests.

We expected that if any comparison between the stimuli and self-produced chemicals took place, this would likely be shown by both TF towards the swab and ground within their home enclosure. Their home enclosure is saturated with their own odour making it possible to detect “self-directed” behaviour through ground-directed TF. These ground-directed TFs were very pronounced and easy to score because animals would always turn their heads away from the swab before tongue-flicking the ground. Although male desert iguanas showed self-directed TF towards their femoral glands (Albert, 1992), we did not expect to find such behaviour in our geckos because we have never observed them turning their body to tongue-flick themselves. What we observed are ground-directed TF when returned to their home enclosure after being removed for 20–30 min. We, indeed, found the expected alternate TF behaviour in our experiment. We recorded higher rates of ground-directed TF compared to swab-directed TF in the unfamiliar condition possibly demonstrating a need for increased comparison with their own odour. We also observed more ground-directed TF when presented with unfamiliar odour than their own. Interestingly, a study in male Iberian rock-lizards (*Lacerta monticola*) showed no differences in non-swab-directed TF between males own and unfamiliar males’ femoral gland secretion (Aragón et al. 2001). This study tested wild caught males that were kept together with a second individual on their reaction to femoral gland secretions. We used chemicals from skin and scats from captive bred individuals kept singly and mainly analysed the reaction from females to these stimuli. It is possible that the scent of the second individual present in the enclosure interfered with “self-directed” TFs in rock-lizards. A comparison to our results is, however, difficult due to the many differences between studies. When individuals are not tested in an environment saturated with their own odour, it is difficult to quantify self-directed behaviour (Aguilar et al. 2009; Cooper et al. 1999; Martin et al. 2020; but see Alberts 1992). Self-directed behaviour might, therefore, be more common in lizards than previously assumed. Contrary, ground-directed TF might not necessarily demonstrate the intent of comparison but rather a way to cleanse their pallet. However, due to the observation of ground-directed TF after return to the home enclosure, we believe that ground-directed TF do show the intent of taking up chemicals produced by themselves.

Our results demonstrate tokay geckos’ ability to discriminate self from other based on skin and scat chemicals. We could also demonstrate that they compare their own odour to a chemical stimulus presented on swabs by tongue-flicking both the swab and ground. This behaviour of tongue-flicking the ground, when interpreted as self-directed behaviour, fulfils one criterion of true self-recognition. Our study, however, lacks certain control conditions. First, we cannot rule out that habituation caused the decreased TF rate (swab and ground TF) when confronted with their own odour. Habituation could be ruled out if lizards continue to show similar low responses when tested in a clean environment. Second, familiarity with their own odour is another potential explanation for the low TF rate. To rule out familiarity, lizards could be familiarised with the odour of a same-sex conspecific first and their responses compared to those towards their own chemicals. Third, lizards’ reaction towards a change in their own odour similar to what was done with dogs (Horowitz 2017) should also be tested. Dogs are more interested in their own odour when it was marked but where less interested in the mark alone. If geckos similarly increase ground-directed TFs towards their marked odour compared to the mark alone then this would show an increased need for comparison and would further support our geckos’ ability to show true self-recognition. Fourth, we already have some evidence that geckos use ground-directed TF to take up their own scent based on the observation that this behaviour is elicited when returning to their enclosure after a time away but this should also be investigated in a systematic manner to show conclusive evidence. We can rule out that diet caused the difference in response towards own and unfamiliar odour because all our lizards were fed the same diet. Novelty does also not seem to explain the change in TF rate as the peppermint oil elicited less TF overall compared to conspecific odour (skin and scat). Overall, our results provide some evidence necessary for true self-recognition but further studies are necessary to rule out alternative explanations for the behaviour we observed.

Finally, our results also point towards a possible social function of scat piling as lizards showed similarly strong responses as well as a similar pattern of swab- and ground-directed TF towards both skin and scat chemicals. Thick-tailed geckos (*Nephrurus milii*) recognise their own scats to add additional faecal matter (Carpenter and Duvall 1995) and social skinks use scat chemicals for group recognition (Bull et al. 2000). Additional research should determine if tokay geckos inspect scat piles of other individuals, if they are more likely to defecate in locations with their own scat present (Carpenter and Duvall 1995), and could investigate if lipids are deposited on scats by glands (Bull et al. 1999b). Furthermore, scat piling might have a possible function related to predator avoidance when predators use the odour of scats to locate refuges (Bull et al. 1999a; Carpenter and Duvall 1995; Norris and Lopez 2011). Studies on wild lizards should document the location of scat piles to determine if scat piles have an anti-predator function as well. Scat piles in locations that are not frequently visited by geckos would point towards an anti-predator function. Such research would provide additional support for a social function of scat piles in tokay geckos.

## Conclusions

In summary, we provide evidence that tokay gecko can discriminate their own chemicals from those produced by same-sex conspecifics and a possible social function of scat piles. Further investigations are, however, needed to confirm true self-recognition and to better understand the communicative function of scats. Future studies could also look at other forms of recognition such as discrimination between familiar and unfamiliar individuals, mate recognition and kin recognition of skin, femoral gland and scat odours. Tokay geckos are a good model species to investigate chemical recognition in general as they show prolonged pair association, biparental care and form temporary family groups with their offspring (Grossmann 2006; Somma 2003). Such studies can potentially demonstrate more sophisticated social cognitive abilities than have previously been attributed to reptiles (Doody et al. 2013). Furthermore, demonstrating true self-recognition in a lizard species would be a first step towards demonstrating self-awareness (Gallup 1998) in reptiles which would provide further evidence that self-awareness is likely widespread across the animal kingdom and might have even been present in a common ancestor.

## Supplementary Information

Below is the link to the electronic supplementary material.

Supplementary file1 (DOCX 516 KB)

Supplementary file2 (MP4 24882 KB)

## Data Availability

All data produced during this study are available on the Open Science Framework (OSF; 10.17605/OSF.IO/JP7H8).

## Code availability

All code used for analysis in R (version 4.0.3, R Core Team, 2020) are available on the Open Science Framework (OSF; 10.17605/OSF.IO/JP7H8).
