# Supplementary material for: Geckos differentiate self from other using both skin and faecal chemicals: evidence towards self-recognition?
Source: Anim Cogn. 2023 Feb 8;26(3):1011–9. doi: 10.1007/s10071-023-01751-8 (PMC10066140; doi:10.1007/s10071-023-01751-8)
Supplement: Supplementary file 1 — Supplementary file1 (DOCX 516 KB) [file 10071_2023_1751_MOESM1_ESM.docx]

# Supplementary material

for the article

“**Geckos differentiate self from other using both skin and faecal chemicals: evidence towards self-recognition?**” by Birgit Szabo and Eva Ringler

from the Division of Behavioural Ecology, Institute of Ecology and Evolution, University of Bern, Bern, Switzerland

Correspond to: Birgit Szabo, Division of Behavioural Ecology, University of Bern, 3032 Bern, Switzerland; email: [birgit.szabo@gmx.at](mailto:birgit.szabo@gmx.at),

## Figures


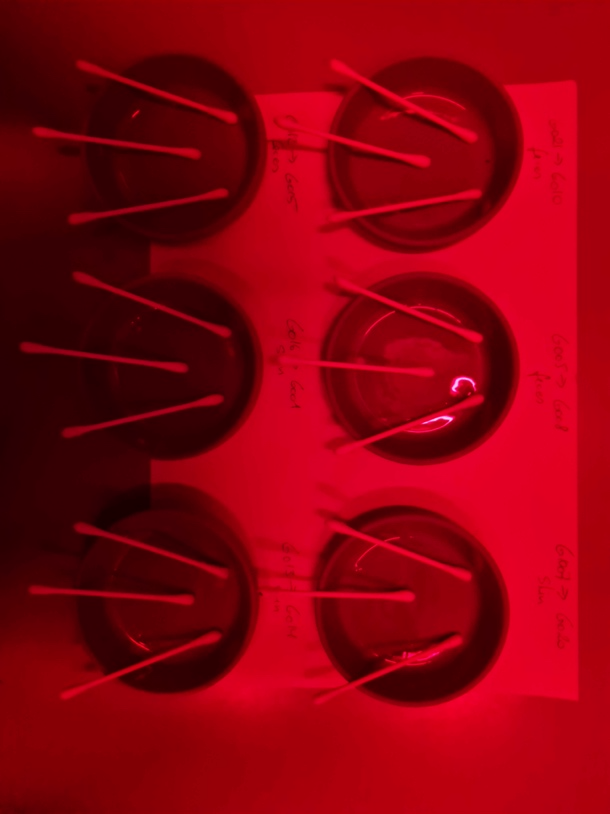


**Figure S1.** Setup of cotton swabs in clay bowls. For each focal subject swabs were placed in a separate clay bowl. Swabs were placed in the testing order. The experimenter made sure that swab tips covered in chemical stimuli never touched each other. To prevent excessive degradation of stimuli, individuals within a room were divided into two groups and the second group set up after the first group finished testing.

## Results tables

**Table S1.** Parameter estimates and test statistics for the generalised linear mixed zero-inflation negative Binomial model looking at tongue flicks produced by all tested individuals in the water control across treatments (pungency control, scat and skin) as well as the least-squares means comparison across treatments. The model included a random effect of animal identity, an over-dispersion parameter of session and an offset of trial time. Significant results are highlighted in bold. CI – confidence interval.

| **Conditional model** | | | | | |
| --- | --- | --- | --- | --- | --- |
| **Parameter** | **Estimate** | ***z*-value** | **CI_low_** | **CI_up_** | ***p*-value** |
| Intercept | 537.773 | 2.232 | 65.651 | 1009.895 | 0.256 |
| Skin | 26.432 | 0.951 | -28.027 | 80.892 | 0.342 |
| Scat | 30.773 | 1.286 | -16.110 | 77.656 | 0.198 |
| **Male** | **-47.915** | **-2.744** | **-82.139** | **-13.961** | **0.006** |
| Room 5 | 4.727 | 0.336 | -22.824 | 32.277 | 0.737 |
| Testing order | 0.592 | 0.653 | -1.184 | 2.367 | 0.514 |
| **Temperature** | **-22.802** | **-2.191** | **-43.196** | **-2.408** | **0.028** |
| **Zero-inflation model** | | | | | |
| **Parameter** | **Estimate** | ***z*-value** | **CI_low_** | **CI_up_** | ***p*-value** |
| Intercept | -0.357 | -0.217 | -3.589 | 2.874 | 0.828 |
| Skin | 0.654 | 0.400 | -2.545 | 3.852 | 0.689 |
| Scat | 0.065 | 0.048 | -2.568 | 2.698 | 0.961 |
| Male | 3.264 | 1.744 | -0.405 | 6.933 | 0.081 |
| **Dispersion model** | | | | | |
| **Intercept** | **-3.647** | **-5.254** | **-5.007** | **-2.286** | **1.49*10^-7^** |
| Session | -0.057 | -0.381 | -0.350 | 0.236 | 0.703 |
| **Least-squares means** | | | | | |
| **Contrasts** | | **Estimate** | **Standard error** | ***t*-ratio** | ***p*-value** |
| Peppermint | Skin | -26.43 | 27.79 | -0.951 | 0.609 |
| Peppermint | Scat | -30.77 | 23.92 | -1.286 | 0.405 |
| Skin | Scat | -4.34 | 8.18 | -0.530 | 0.857 |

**Table S2.** Parameter estimates and test statistics for the generalised linear mixed zero-inflation negative Binomial model looking at all tongue flicks produced by females as well as the least-squares means comparison across treatments. The model included a random effect of animal identity, an over-dispersion parameter of session and an offset of trial time. Significant results are highlighted in bold. CI – confidence interval.

| **Conditional model** | | | | | |
| --- | --- | --- | --- | --- | --- |
| **Parameter** | **Estimate** | ***z*-value** | **CI_low_** | **CI_up_** | ***p*-value** |
| **Intercept** | **228.182** | **2.289** | **32.759** | **423.604** | **0.022** |
| **Skin** | **17.035** | **2.385** | **3.033** | **31.037** | **0.017** |
| **Scat** | **18.916** | **3.165** | **7.201** | **30.631** | **0.002** |
| Stimulus order | 2.811 | 1.853 | -0.163 | 5.785 | 0.064 |
| Testing order | 0.249 | 0.779 | -0.377 | 0874 | 0.436 |
| Trial | 0.874 | 0.585 | -2.055 | 3.802 | 0.559 |
| Room 5 | 6.380 | 1.285 | -3.350 | 16.110 | 0.198 |
| **Temperature** | **-10.600** | **-2.521** | **-18.840** | **-2.360** | **0.012** |
| **Zero-inflation model** | | | | | |
| **Parameter** | **Estimate** | ***z*-value** | **CI_low_** | **CI_up_** | ***p*-value** |
| Intercept | -2.636 | -1.329 | -6.524 | 1.251 | 0.184 |
| Skin | 1.996 | 1.772 | -1.477 | 5.468 | 0.260 |
| Scat | 1.136 | 1.745 | -2.283 | 4.556 | 0.515 |
| **Dispersion model** | | | | | |
| **Intercept** | **-3.887** | **-11.270** | **-4.563** | **-3.210** | **< 2*10^-16^** |
| Session | -0.037 | -0.540 | -0.173 | 0.098 | 0.589 |
| **Least-squares means** | | | | | |
| **Contrasts** | | **Estimate** | **Standard error** | ***t*-ratio** | ***p*-value** |
| **Peppermint** | **Skin** | **-17.030** | **7.140** | **-2.385** | **0.047** |
| **Peppermint** | **Scat** | **-18.920** | **5.980** | **-3.165** | **0.005** |
| Skin | Scat | -1.880 | 2.990 | -0.629 | 0.804 |

**Table S3.** Parameter estimates and test statistics for the generalised linear mixed zero-inflation negative Binomial model looking at all tongue flicks produced in the unfamiliar condition by all tested individuals in response to delta SVL. The model included animal identity as the random effect, an over-dispersion parameter of session and an offset of trial time. Significant results are highlighted in bold. CI – confidence interval.

| **Conditional model** | | | | | |
| --- | --- | --- | --- | --- | --- |
| **Parameter** | **Estimate** | ***z*-value** | **CI_low_** | **CI_up_** | ***p*-value** |
| **Intercept** | **-11.403** | **-8.713** | **-13.969** | **-8.838** | **< 2*10^-16^** |
| Delta SVL | -0.034 | -0.031 | -2.184 | 2.117 | 0.976 |
| **Dispersion model** | | | | | |
| **Parameter** | **Estimate** | ***z*-value** | **CI_low_** | **CI_up_** | ***p*-value** |
| **Intercept** | **-4.119** | **-7.961** | **-5.133** | **-3.105** | **1.7*10^-15^** |
| Session | -0.101 | -0.923 | -0.316 | 0.114 | 0.356 |

**Table S4.** Parameter estimates and test statistics for the linear mixed model looking at deep breaths per second. The model included session and animal identity as random effects. CI – confidence interval.

| **Parameter** | **Estimate** | ***t*-value** | **CI_low_** | **CI_up_** | ***p*-value** |
| --- | --- | --- | --- | --- | --- |
| Intercept | 0.529 | 0.428 | -1.745 | 2.841 | 0.671 |
| Skin | 0.026 | 0.230 | -0.180 | 0.229 | 0.821 |
| Scat | 0.041 | 0.364 | -0.165 | 0.244 | 0.721 |
| Water control | 0.023 | 0.723 | -0.038 | 0.085 | 0.470 |
| Own odour | -0.005 | -0.165 | -0.066 | 0.057 | 0.869 |
| Pungency control | -0.057 | -0.668 | -0.223 | 0.109 | 0.505 |
| Male | 0.107 | 1.958 | 0.003 | 0.212 | 0.066 |
| Stimulus order | -0.018 | -1.130 | -0.048 | 0.013 | 0.259 |
| Trial | -0.024 | -1.108 | -0.065 | 0.024 | 0.275 |
| Temperature | -0.009 | -0.180 | -0.105 | 0.084 | 0.858 |
| Room 5 | 0.009 | 0.150 | -0.105 | 0.124 | 0.882 |

**Table S5.** Parameter estimates and test statistics for the linear mixed model looking at breaths per second. The model included a random intercept of animal identity and a random slope of session. Significant results are highlighted in bold. CI – confidence interval.

| **Parameter** | **Estimate** | ***t*-value** | **CI_low_** | **CI_up_** | ***p*-value** |
| --- | --- | --- | --- | --- | --- |
| Intercept | 0.884 | 0.684 | -1.633 | 3.341 | 0.495 |
| Skin | -0.007 | -0.047 | -0.285 | 0.271 | 0.962 |
| Scat | 0.100 | 0.716 | -0.178 | 0.375 | 0.475 |
| Water control | 0.043 | 1.161 | -0.030 | 0.115 | 0.248 |
| Own odour | 0.020 | 0.533 | -0.053 | 0.095 | 0.595 |
| Pungency control | -0.001 | -0.009 | -0.267 | 0.269 | 0.993 |
| Male | 0.008 | 0.154 | -0.096 | 0.114 | 0.880 |
| Stimulus order | -0.010 | -0.545 | -0.047 | 0.026 | 0.586 |
| Trial | -0.037 | -1.737 | -0.079 | 0.006 | 0.088 |
| Temperature | 0.003 | 0.057 | -0.099 | 0.107 | 0.955 |
| Room | 0.053 | 0.850 | -0.064 | 0.169 | 0.402 |

**Table S6.** Parameter estimates and test statistics for the generalised linear mixed zero-inflation negative Binomial model looking at swab and ground directed tongue flicks across stimulus conditions in females. The conditional model included trial and animal identity as random effects, an over-dispersion parameter of session and an offset of trial time; no zero-inflation was specified. Significant results are highlighted in bold. TFg – ground directed TF, CI – confidence interval.

| **Conditional model** | | | | | | | |
| --- | --- | --- | --- | --- | --- | --- | --- |
| **Parameter** | | **Estimate** | | ***z*-value** | **CI_low_** | **CI_up_** | ***p*-value** |
| **Intercept** | | **616.832** | | **5.907** | **412.159** | **821.506** | **3.49*10^-9^** |
| **Own odour** | | **-19.220** | | **-3.339** | **-30.502** | **-7.937** | **0.0008** |
| **Unfamiliar odour** | | **-10.719** | | **-3.447** | **-32.501** | **-8.938** | **0.0006** |
| Ground TF | | 0.319 | | 0.070 | -8.586 | 9.224 | 0.944 |
| **Temperature** | | **-24.994** | | **-6.272** | **-32.805** | **-17.183** | **3.57*10^-10^** |
| Interaction:  Own odour * TFg | | 2.015 | | 0.258 | -13.318 | 17.348 | 0.797 |
| **Interaction:**  **Unfamiliar odour * TFg** | | **33.651** | | **4.790** | **19.882** | **47.420** | **1.67*10^-6^** |
| **Dispersion model** | | | | | | | |
| **Parameter** | | **Estimate** | | ***z*-value** | **CI_low_** | **CI_up_** | ***p*-value** |
| **Intercept** | | **-4.246** | | **-15.488** | **-4.783** | **-3.708** | **<2*10^-16^** |
| **Session** | | **-0.134** | | **-2.269** | **-0.251** | **-0.018** | **0.023** |
| **Least-squares means – comparison of ground and swab directed TF within stimuli** | | | | | | | |
| **Contrasts** | | | **Estimate** | | **Standard error** | ***t*-ratio** | ***p*-value** |
| **Water control** | | | | | | | |
| Swab TF | Ground TF | | -0.319 | | 4.540 | -0.070 | 0.944 |
| **Own odour** | | | | | | | |
| Swab TF | Ground TF | | -2.334 | | 6.250 | -0.373 | 0.709 |
| **Unfamiliar odour** | | | | | | | |
| **Swab TF** | **Ground TF** | | **-33.970** | | **4.950** | **-6.860** | **0.0001** |
| **Least-squares means – comparison of ground directed TF across stimuli and swab directed TF across stimuli** | | | | | | | |
| **Swab directed TF** | | | | | | | |
| **Water control** | **Own** | | **19.2** | | **5.76** | **3.339** | **0.003** |
| **Water control** | **Unfamiliar** | | **20.7** | | **6.01** | **3.447** | **0.002** |
| Own | Unfamiliar | | 1.5 | | 4.42 | 0.339 | 0.939 |
| **Ground directed TF** | | | | | | | |
| **Water control** | **Own** | | **17.2** | | **4.64** | **3.710** | **0.0007** |
| **Water control** | **Unfamiliar** | | **-12.9** | | **4.73** | **-2.735** | **0.018** |
| **Own** | **Unfamiliar** | | **-30.1** | | **5.28** | **-5.709** | **0.0001** |
